# The Impacts of *msaABCR* on *sarA*-Associated Phenotypes Are Different in Divergent Clinical Isolates of Staphylococcus aureus

**DOI:** 10.1128/IAI.00530-19

**Published:** 2020-01-22

**Authors:** Joseph S. Rom, Aura M. Ramirez, Karen E. Beenken, Gyan S. Sahukhal, Mohamed O. Elasri, Mark S. Smeltzer

**Affiliations:** a Department of Microbiology and Immunology, University of Arkansas for Medical Sciences, Little Rock, Arkansas, USA; b Department of Orthopaedic Surgery, University of Arkansas for Medical Sciences, Little Rock, Arkansas, USA; c Center for Molecular and Cellular Biosciences, University of Southern Mississippi, Hattiesburg, Mississippi, USA; University of Illinois at Chicago

**Keywords:** LAC, *Staphylococcus aureus*, UAMS-1, biofilms, global regulatory networks, *msa*, nucleases, osteomyelitis, proteases, *sarA*

## Abstract

The staphylococcal accessory regulator (*sarA*) plays an important role in Staphylococcus aureus infections, including osteomyelitis, and the *msaABCR* operon has been implicated as an important factor in modulating expression of *sarA*. Thus, we investigated the contribution of *msaABCR* to *sarA*-associated phenotypes in the S. aureus clinical isolates LAC and UAMS-1. Mutation of *msaABCR* resulted in reduced production of SarA and a reduced capacity to form a biofilm in both strains. Biofilm formation was enhanced in a LAC *msa* mutant by restoring the production of SarA, but this was not true in a UAMS-1 *msa* mutant. Similarly, extracellular protease production was increased in a LAC *msa* mutant but not a UAMS-1 *msa* mutant. This difference was reflected in the accumulation and distribution of secreted virulence factors and in the impact of extracellular proteases on biofilm formation in a LAC *msa* mutant. Most importantly, it was reflected in the relative impact of mutating *msa* as assessed in a murine osteomyelitis model, which had a significant impact in LAC but not in UAMS-1. In contrast, mutation of *sarA* had a greater impact on all of these *in vitro* and *in vivo* phenotypes than mutation of *msaABCR*, and it did so in both LAC and UAMS-1. These results suggest that, at least in osteomyelitis, it would be therapeutically preferable to target *sarA* rather than *msaABCR* to achieve the desired clinical result, particularly in the context of divergent clinical isolates of S. aureus.

## Abstract (precis)

The staphylococcal accessory regulator (*sarA*) plays an important role in Staphylococcus aureus infections, including osteomyelitis, and the *msaABCR* operon has been implicated as an important factor in modulating expression of *sarA*. Thus, we investigated the contribution of *msaABCR* to *sarA*-associated phenotypes in the S. aureus clinical isolates LAC and UAMS-1. Mutation of *msaABCR* resulted in reduced production of SarA and a reduced capacity to form a biofilm in both strains.

## INTRODUCTION

Mutation of the staphylococcal accessory regulator (*sarA*) attenuates the virulence of divergent clinical isolates of Staphylococcus aureus in animal models of bacteremia, postsurgical osteomyelitis, and infective endocarditis (1–3). It also limits biofilm formation *in vitro* and *in vivo* to a degree that can be correlated with increased antibiotic susceptibility (2, 4–6). The effector molecule of the *sarA* regulatory system is a 15-kDa protein that has been shown to impact the production of multiple S. aureus virulence factors at a transcriptional level and by modulating the stability of mRNA (7–12). We have also demonstrated that an important factor contributing to the reduced virulence of *sarA* mutants, and their reduced capacity to form a biofilm, is the increased production of extracellular proteases and resulting decrease in the accumulation of multiple S. aureus proteins, including both surface-associated and extracellular virulence factors (1, 13–17).

Thus, the *sarA* regulatory locus impacts both the production and the accumulation of S. aureus virulence factors, and this collectively makes an important contribution to diverse phenotypes that contribute to pathogenesis. This makes *sarA* a potential therapeutic target, and efforts have been made to exploit *sarA* in this regard (17–19). However, S. aureus regulatory circuits are complex and highly interactive (20), and mutation of other S. aureus regulatory loci within this circuit has also been shown to increase protease production to a degree that limits biofilm formation (21–25).

Among these other loci is *msa* (modulator of *sarA*), mutation of which was originally reported to limit the expression of *sarA* and the production of SarA itself (26). The *msa* gene was identified in the 8325-4 strain RN6390 by a transposon insertion in the open-reading frame SA1233 as designated in the N315 genome, but it was subsequently shown to be part of a four-gene operon now designated *msaABCR* (27). Genes within the *msa* operon encode a putative protein (MsaA) with no known function, a DNA binding protein (MsaB) shown to act as a transcription factor that regulates expression of numerous genes, and a regulatory RNA (*msaC*) and an antisense RNA (*msaR*) complementary to *msaB* (27). As would be expected based on the phenotypes of *sarA* mutants (3, 4, 13, 15, 16, 28) and the role of *msaABCR* in enhancing expression of *sarA*, mutation of *msaABCR* (hereinafter referred to as *msa*) has been correlated with increased protease production and a decreased capacity to form a biofilm (25, 27, 29).

Mutation of *msa* was also reported to result in decreased expression of the accessory gene regulator (*agr*) in the 8325-4 strain RN6390 but to have the opposite effect in the clinical isolate UAMS-1 (26). Expression levels of the well-characterized *agr*-regulated genes encoding alpha toxin (*hla*) and protein A (*spa*) also differed between these two strains, while expression of the genes encoding aureolysin (*aur*) and SspA (*sspA*) was increased in both strains. Differences between these two strains have also been observed in the phenotype of their isogenic *sarA* mutants (30, 31). Such reports are not surprising given that RN6390 has a mutation in *rsbU* that impacts the *sigB* regulatory pathway (32), which has also been shown to impact expression of both *agr* and *sarA* as well as protease production (33, 34). However, significant differences also exist among clinical isolates, and to date, such strain-dependent differences have not been adequately investigated. Thus, the overall impact of *msa* in divergent clinical isolates, and the extent to which it is dependent on its interaction with *sarA*, remains unclear. In this report, we addressed these issues by generating *msa*, *sarA*, and *msa sarA* mutants in the methicillin-resistant USA300 strain LAC and the methicillin-sensitive USA200 strain UAMS-1 and assessed the impact these mutations had on well-defined phenotypes associated with their isogenic *sarA* mutants.

## RESULTS AND DISCUSSION

### Impacts of *msa* on *sarA* expression.

**FIG 1:**
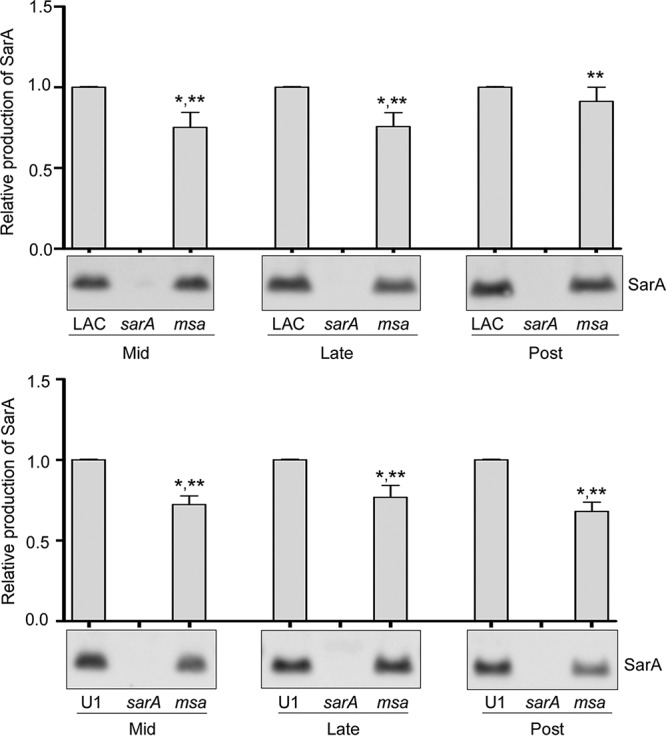
Impact of *msa* on the accumulation of SarA. SarA accumulation was assessed by Western blotting of whole-cell lysates prepared from mid-, late-, or post-exponential-phase cultures of LAC, UAMS-1 (U1), and their isogenic *msa* and *sarA* mutants. Bar charts illustrate densitometry based on two biological replicates. Densitometry results from samples prepared from each parent strain using cells obtained at each growth phase were standardized to OD_560_ of 10. Error bars indicate standard errors of the means. *, statistical significance relative to the isogenic parent strain; **, statistical significance relative to the isogenic *sarA* mutant.

Using an anti-SarA antibody (35), we first assessed the production of SarA in *msa* mutants generated in LAC and UAMS-1 by Western blotting. Experiments were performed using whole-cell lysates prepared from equal numbers of CFU harvested from cultures in the mid-, late-, and post-exponential growth phases. The results were comparable in both strains (Fig. 1) and confirmed that mutation of *msa* results in reduced production of SarA, particularly during the mid- and late-exponential growth phases. However, while the differences in the abundance of SarA were in most cases statistically significant, they were also modest in that the amount of SarA present in lysates prepared from LAC and UAMS-1 *msa* mutants was consistently >50% of that observed in the isogenic parent strain irrespective of growth stage. This is consistent with transcriptional analysis, which demonstrated that mutation of *msa* results in a modest but statistically significant decrease in the levels of *sarA* transcripts in both LAC and UAMS-1 compared to that in the isogenic parent strain (Table 1). These studies also confirmed that this transcriptional phenotype could be genetically complemented. These results are consistent with the hypothesis that *msa* functions upstream to modulate the expression of SarA.

**TABLE 1:** *sarA* expression at mid-exponential growth phase

Strain	Expression relative to WT^*a*^
LAC Δ*msaABCR*	0.493 ± 0.01
LAC Δ*msaABCR*/pCN34::*msaABCR*	0.984 ± 0.0168
UAMS-1 Δ*msaABCR*	0.753 ± 0.016
UAMS-1 Δ*msaABCR*/pCN34::*msaABCR*	0.875 ± 0.019

a WT, wild type.

### Impact of *msa* on biofilm formation.

**FIG 2:**
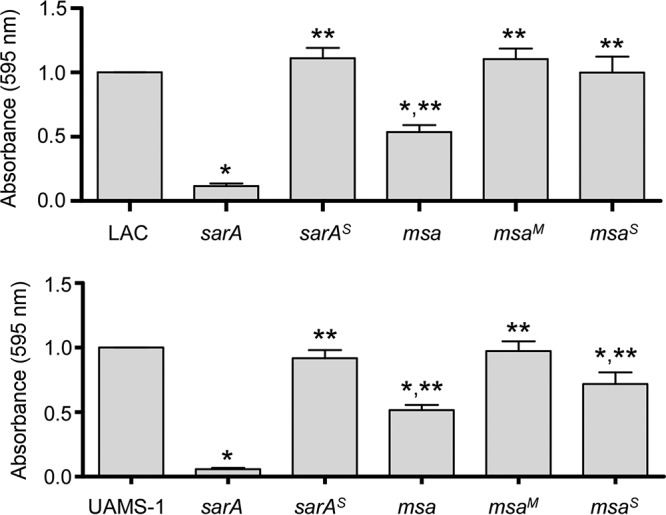
Impacts of *msa* and *sarA* on biofilm formation. Biofilm formation was assessed with LAC, UAMS-1, their *sarA* and *msa* mutants, and mutants complemented with *sarA* (*^S^*) or *msa* (*^M^*). Bar charts represent cumulative results from at least two biological replicates, each of which included five experimental replicates. Error bars indicate standard errors of the means. *, statistical significance relative to the isogenic parent strain; **, statistical significance relative to the isogenic *sarA* mutant.

Thus, the important question becomes whether the reduction in the amount of SarA observed in *msa* mutants is phenotypically relevant. One of the primary phenotypes that define *sarA* mutants in divergent clinical isolates, including LAC and UAMS-1, is the reduced capacity to form a biofilm (36). Using a well-established microtiter plate assay (28), we confirmed that mutation of *msa* limits biofilm formation in both LAC and UAMS-1 but to a limited extent compared to that of the isogenic *sarA* mutants (Fig. 2). The relative impact of mutating *msa* versus *sarA* was confirmed by demonstrating that concomitant mutation of both *msa* and *sarA* limited biofilm formation to a level comparable to that observed in the isogenic *sarA* mutant and well below that observed in the corresponding *msa* mutant (see Fig. S1 in the supplemental material). These results are also consistent with the hypothesis that *msa* is upstream of SarA and the observation that mutation of *msa* had only a modest impact on the accumulation of SarA, but they also suggest that the reduced amount of SarA observed in *msa* mutants is phenotypically relevant in the context of biofilm formation.

**FIG 3:**
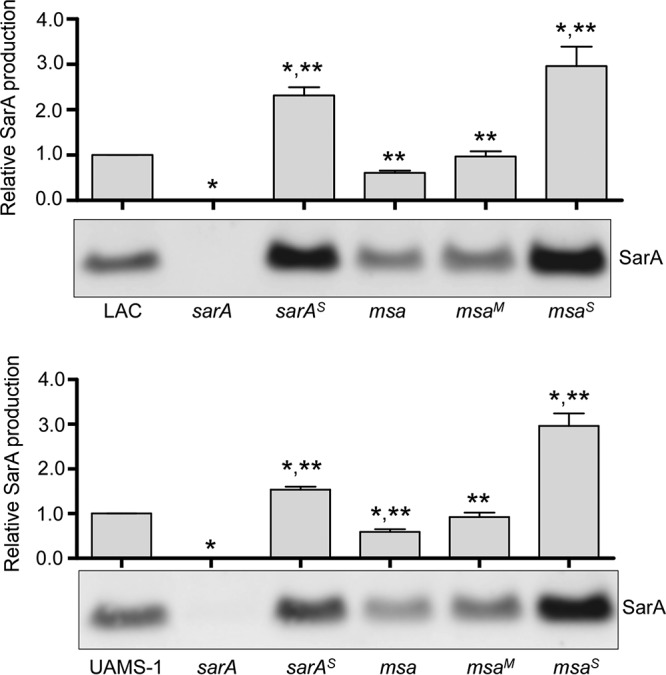
SarA accumulation in *sarA*- and *msa*-complemented mutants. SarA accumulation was assessed by Western blotting of whole-cell lysates prepared from mid-exponential-phase cultures of LAC, UAMS-1, their *sarA* and *msa* mutants, and mutants complemented with *sarA* (*^S^*) or *msa* (*^M^*). Bar charts illustrate densitometry based on at least two experimental replicates. Densitometry was performed using samples prepared from cells obtained at mid-exponential growth phase (standardized to OD_560_ of 1.5). Error bars indicate standard errors of the means. *, statistical significance relative to the isogenic parent strain; **, statistical significance relative to the isogenic *sarA* mutant.

If this is true, then restoring the production of SarA in an *msa* mutant should restore biofilm formation. To investigate this, we introduced the same plasmid (pSARA) used to genetically complement the *sarA* mutation into an *msa* mutant. Western blot analysis confirmed that the accumulation of SarA was restored in both LAC and UAMS-1 *msa* mutants (Fig. 3). Introducing pSARA also restored biofilm formation in a LAC *msa* mutant but not in a UAMS-1 *msa* mutant (Fig. 2). The reasons for this strain-dependent difference are unclear, but these results suggest that *msa* limits biofilm formation in UAMS-1 owing to a *sarA*-independent regulatory effect.

### Impact of *msa* on protease production.

**FIG 4:**
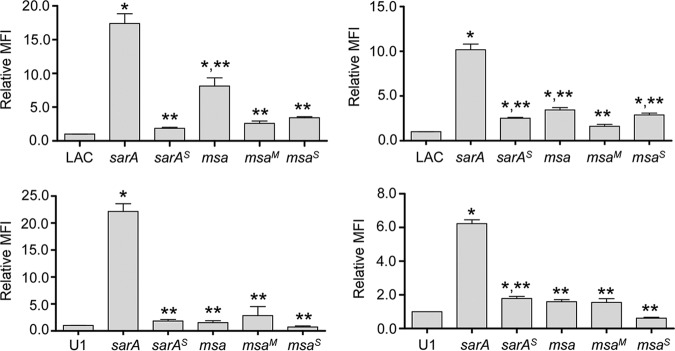
Impacts of *msa* and *sarA* on protease production. Protease activity in conditioned medium (CM) was assessed with LAC, UAMS-1, their *sarA* and *msa* mutants, and mutants complemented with *sarA* (*^S^*) or *msa* (*^M^*). Protease activity was assessed using a fluorescein isothiocyanate (FITC)-casein cleavage hydrolysis assay (left) and a FITC-gelatin cleavage hydrolysis assay (right). Results are reported as mean fluorescence values (MFIs) ± the standard errors of the means. Bar charts are representative of results from at least two biological replicates, each of which included three experimental replicates. *, statistical significance relative to the isogenic parent strain; **, statistical significance relative to the isogenic *sarA* mutant.

To investigate the mechanistic basis for these biofilm phenotypes, we examined the relative impact of mutating *sarA* and *msa* on the production of extracellular proteases. This was based on our previous demonstration that the increased production of extracellular proteases plays a key role in defining the biofilm-deficient phenotype of S. aureus *sarA* mutants (1). In LAC, mutation of *msa* resulted in a statistically significant increase in overall protease activity as assessed using both casein- and gelatin-based fluorescence resonance energy transfer (FRET) assays, although the impact was more evident in the casein-based assay than in the gelatin-based assay (Fig. 4). This was not true in a LAC *sarA* mutant, where the impact of mutating *sarA* on protease production was readily evident in both assays (Fig. 4). Additionally, restoring SarA production in a LAC *msa* mutant decreased protease production, in the case of the casein-based assay, to wild-type levels. As might be expected based on the relative sensitivity of the two assays, this was most evident when assessed using the casein-based assay. However, mutation of *msa* in UAMS-1 did not have a significant impact on overall protease activity as assessed using either casein- or gelatin-based FRET assays (Fig. 4). As in LAC, mutation of *sarA* in UAMS-1 resulted in a statistically significant increase in protease production in both protease assays. These results are also consistent with the hypothesis that the impact of mutating *msa* on biofilm formation in UAMS-1 occurs via a *sarA*-independent regulatory effect.

**FIG 5:**
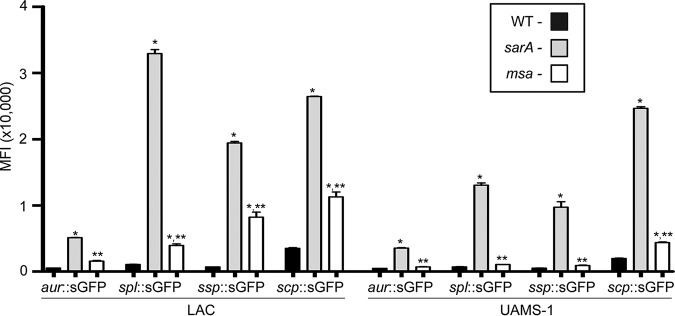
Impacts of *msa* and *sarA* on protease gene expression. Reporter constructs were generated using the promoters from each of the four genes/operons encoding extracellular proteases and the gene encoding green fluorescent protein (*gfp*). Each construct was introduced into LAC, UAMS-1, and their isogenic *sarA* and *msa* mutants. Mean fluorescence intensity (MFI) was assessed after overnight cultures standardized to an OD_560_ of 10. Bars represent average MFIs ± standard errors of the means from two independent biological replicates, each of which included three experimental replicates. Statistical analysis was performed independently for each strain and each reporter. *, statistical significance compared to the isogenic parent strain; **, statistical significance compared to the isogenic *sarA* mutant.

This strain-dependent difference was also apparent in assays employing *gfp* transcriptional reporter constructs generated with the promoters from each of the genes and/or operons encoding S. aureus extracellular proteases (*aur*, *splA-F*, *sspABC*, and *scpAB*). Specifically, expression levels from all four reporters were significantly increased in a LAC *msa* mutant but not to the level observed in the isogenic *sarA* mutant (Fig. 5). In contrast, fluorescence was not increased to a significant extent in a UAMS-1 *msa* mutant with any reporter other than the *scp*::*gfp*, and even then, the increase was modest by comparison to fluorescence levels observed with the same reporter in the LAC *msa* mutant and with all four reporters in the UAMS-1 *sarA* mutant (Fig. 5). These results suggest that the strain-dependent impact of *msa* on protease production is mediated at a transcriptional level.

**FIG 6:**
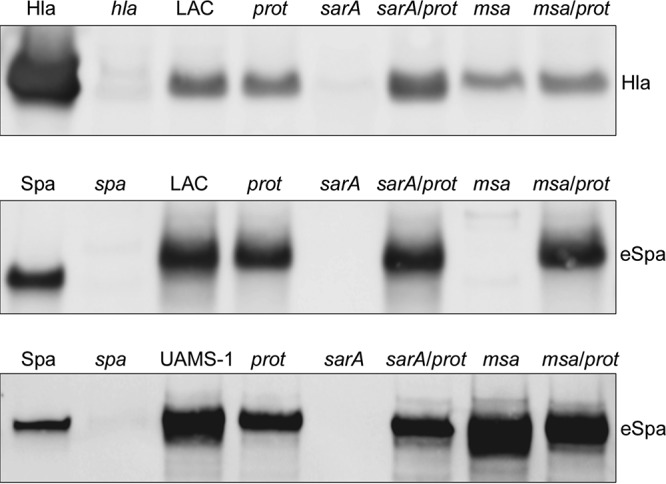
Impact of extracellular proteases on accumulation of specific proteins. The abundance of alpha toxin (Hla) and extracellular protein A (eSpa) was assessed by Western blotting of CM obtained from stationary-phase cultures of LAC and UAMS-1, their *sarA* and *msa* mutants, and isogenic derivatives of each strain unable to produce extracellular proteases (*prot*). Purified Spa and Hla were included as positive controls. CM from LAC *spa* and *hla* mutants were included as negative controls.

These results also suggest the possibility of a cause-and-effect relationship between increased protease production and decreased biofilm formation in a LAC *msa* mutant. Indeed, there was an inverse and proportional relationship between protease production and biofilm formation in LAC and its isogenic *sarA*, *msa*, and *sarA msa* mutants (see Fig. S2). However, this inverse relationship was not apparent in a UAMS-1 *msa* mutant. Mutation of *msa* in LAC also resulted in the decreased accumulation of both Hla and extracellular protein A (eSpa) (Fig. 6). In contrast, in UAMS-1, which does not produce Hla, the accumulation of eSpa was greatly reduced in a *sarA* mutant but not in the isogenic *msa* mutant. The reduced accumulation of eSpa observed in a LAC *msa* mutant was reversed by eliminating the production of extracellular proteases, while in a UAMS-1 *msa* mutant, the abundance of eSpa was not affected by the inability to produce these proteases (Fig. 6).

**FIG 7:**
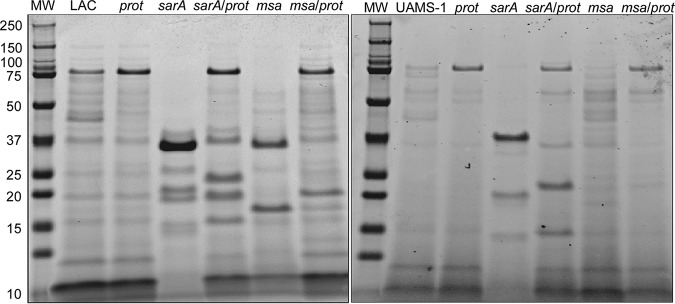
Impacts of *sarA* and *msa* on accumulation of extracellular proteins. Extracellular protein profiles were assessed by SDS-PAGE analysis of CM obtained from stationary-phase cultures of LAC, UAMS-1, their *sarA* and *msa* mutants, and isogenic derivatives of each strain unable to produce extracellular proteases (*prot*). MW, molecular weight marker.

These results demonstrate that mutating *msa* results in a significant increase in protease production in LAC but not in UAMS-1. SDS-PAGE analysis of conditioned medium (CM) from overnight cultures confirmed the decreased accumulation of high-molecular-weight (HMW) proteins in a LAC *msa* mutant and that this was reversed by eliminating the production of extracellular proteases (Fig. 7). As would be expected based on the results discussed above, this effect was not apparent in a UAMS-1 *msa* mutant. In contrast, mutation of *sarA* limited the accumulation of HMW proteins in CM in both LAC and UAMS-1, and in both cases, this was reversed by eliminating the ability of these mutants to produce extracellular proteases (Fig. 7).

### Impact of *msa* on PIA production.

To examine other possibilities, we assessed the production of the polysaccharide intracellular adhesin (PIA) in *msa* and *sarA* mutants. PIA is known to contribute to biofilm formation, and it has been suggested that it plays a particularly important role in methicillin-sensitive strains such as UAMS-1 (37). However, we were unable to detect PIA above background levels in LAC, UAMS-1, or their isogenic *sarA* and *msa* mutants (see Fig. S3).

### Impact of *msa* on extracellular nuclease.

Extracellular DNA and the production of extracellular nucleases have also been implicated in biofilm formation in both methicillin-resistant and methicillin-sensitive strains (38). S. aureus produces at least two nucleases, one of which (Nuc1) is a secreted extracellular protein, while the other (Nuc2) remains bound to the cell surface (39). Mutation of *sarA* in UAMS-1 has been shown to result in the increased production of these nucleases, and at least under *in vitro* conditions, this has been shown to limit biofilm formation (40). Based on this, we examined the impact of mutating *msa* on nuclease production with a specific focus on the Nuc1 extracellular nuclease. This was facilitated by the availability of an anti-Nuc1 antibody (16), which allowed us to investigate this issue using Western blots of CM harvested from overnight cultures of each strain. It is important to recognize that Nuc1 is produced in two forms, the smaller of which (NucA) is proteolytically derived from the larger (NucB), and both of which are enzymatically active (41).

**FIG 8:**
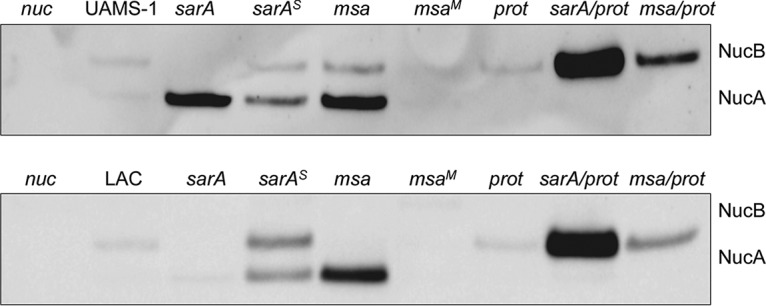
Impacts of proteases on Nuc1 production and processing in *sarA* and *msa* mutants. The amount of extracellular nuclease was assessed by Western blotting using CM from LAC, UAMS-1, their isogenic *sarA* and *msa* mutants, *sarA* (*^S^*) or *msa* (*^M^*) complemented variants, and isogenic derivatives of regulatory mutants unable to produce extracellular proteases (*prot*). A UAMS-1 *nuc1* (*nuc*) mutant was included as a negative control in both blots.

Relative to the parent strain, Nuc1 was present in increased amounts in a UAMS-1 *sarA* mutant, and all of the Nuc1 present that could be detected by Western blotting was present in the smaller NucA form (Fig. 8). This suggests that the increased production of extracellular proteases in a UAMS-1 *sarA* mutant can be correlated with the absence of NucB. This was confirmed in Western blots with CM from a *sarA* mutant unable to produce these proteases, in which case, all of the Nuc1 detected was in the NucB form. Moreover, the overall abundance of Nuc1 was increased in the protease-deficient UAMS-1 *sarA* mutant compared to that in the *sarA* mutant (Fig. 8). The abundance of Nuc1 was also increased in a UAMS-1 *msa* mutant, and in this case, both NucA and NucB were detectable by Western blotting. While the overall amount of Nuc1 was not increased in a protease-deficient UAMS-1 *msa* mutant, all of the Nuc1 present was in the larger NucB form. This could be interpreted to suggest that mutation of *msa* does result in an increase in protease production in UAMS-1 that is phenotypically apparent, but we believe this would be an overinterpretation in that, unlike in the isogenic protease-deficient *sarA* mutant, the amount of Nuc1 did not increase appreciably in the UAMS-1 protease-deficient *msa* mutant (Fig. 8).

The increased abundance of Nuc1 observed in a UAMS-1 *sarA* mutant was not apparent in a LAC *sarA* mutant, but it was apparent in the isogenic *msa* mutant (Fig. 8). Unlike in the UAMS-1 *msa* mutant, all of the Nuc1 detectable by Western blotting in the LAC *msa* mutant was present in the smaller NucA form. This is consistent with the observation that mutating *msa* had a significant impact on protease production in LAC but not in UAMS-1. As with the UAMS-1 protease-deficient *sarA* and *msa* mutants, only NucB was detected in CM from the protease-deficient LAC *sarA* and *msa* mutants (Fig. 8). As with a UAMS-1 *msa* mutant, eliminating protease production in a LAC *msa* mutant limited proteolytic processing of Nuc1 but did not appreciably alter the overall amount. In contrast, the abundance of NucB was also enhanced in a protease-deficient LAC *sarA* mutant compared to that in the isogenic *sarA* mutant itself. These results demonstrate that the production of Nuc1 is increased in LAC and UAMS-1 *sarA* and *msa* mutants. They also indicate that the abundance of Nuc1 is limited by increased protease production in *sarA* mutants generated in both strains but that this is not the case in LAC *msa* mutants. However, the impact of *msa* on protease production was still evident in a LAC *msa* mutant, in that all of the Nuc1 present was present in the smaller NucA form (Fig. 8).

### Impacts of protease and nuclease production on biofilm formation.

**FIG 9:**
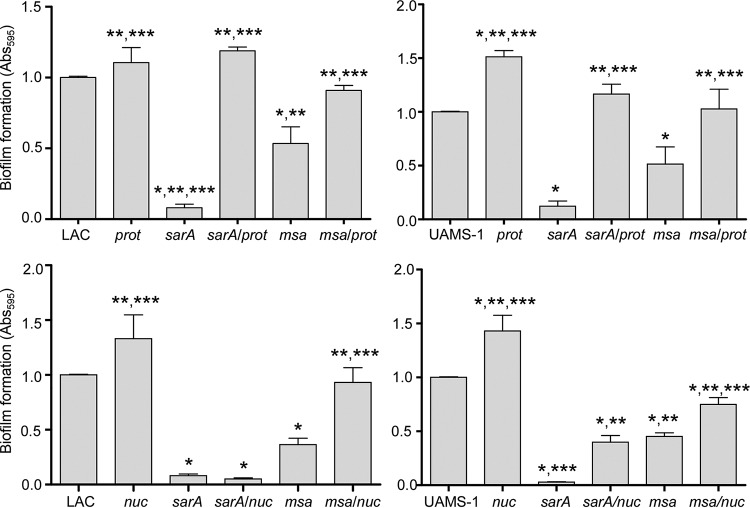
Impacts of extracellular proteases and nucleases on biofilm formation in *msa* and *sarA* mutants. Biofilm formation was assessed with LAC, UAMS-1, their *sarA* and *msa* mutants, and isogenic derivatives of each strain unable to produce either extracellular proteases (*prot*; top) or the extracellular nuclease Nuc1 (*nuc*; bottom). Bar charts indicate cumulative results from at least two biological replicates, each of which included five experimental replicates. Error bars indicate standard errors of the means. *, statistical significance relative to the isogenic parent strain; **, statistical significance relative to the isogenic *sarA* mutant; ***, statistical significance relative to the isogenic *msa* mutant.

Given these overlapping protease and nuclease phenotypes, we directly examined the impacts of eliminating the production of extracellular proteases or Nuc1 on the biofilm-deficient phenotype of LAC and UAMS-1 *sarA* and *msa* mutants. In both strains, eliminating the ability to produce extracellular proteases enhanced biofilm formation in both *sarA* and *msa* mutants to levels comparable to those observed in the isogenic parent strain (Fig. 9). This could be interpreted to suggest that the increased production of extracellular proteases limits biofilm formation in *msa* mutants, even in UAMS-1. However, it is important to note that eliminating protease production also enhanced biofilm formation in UAMS-1 itself to a greater extent than in LAC (Fig. 9). In fact, the increase in biofilm formation observed in a protease-deficient derivative of UAMS-1 was comparable to that observed in the UAMS-1 *msa* mutant, and this was not the case in the same derivatives of LAC. Thus, we believe these results are also consistent with the conclusion that the increased production of extracellular proteases limits biofilm formation in a LAC *msa* mutant but not in a UAMS-1 *msa* mutant.

Biofilm formation was also enhanced in LAC and UAMS-1 *msa* mutants unable to produce Nuc1, but once again, these results must be interpreted with caution, because eliminating the production of Nuc1 also enhanced biofilm formation in the LAC and UAMS-1 parent strains (Fig. 9). As with protease production, the increase in biofilm formation observed in the nuclease-deficient UAMS-1 *msa* mutant was less than that observed in the nuclease-deficient LAC *msa* mutant, and this was reflected in the relative impact of eliminating Nuc1 production on biofilm formation (Fig. 9). In contrast, eliminating the production of Nuc1 did have a significant impact on biofilm formation in a UAMS-1 *sarA* mutant but not in a LAC *sarA* mutant (Fig. 9). This is consistent with the observation that mutation of *msa* resulted in an increase in the abundance of Nuc1 in a UAMS-1 *sarA* mutant but not in a LAC *sarA* mutant, although as previously discussed, protease production was shown to limit the abundance and processing of Nuc1 in *sarA* mutants generated in both strains.

### Impact of *msa* on staphyloxanthin production.

**FIG 10:**
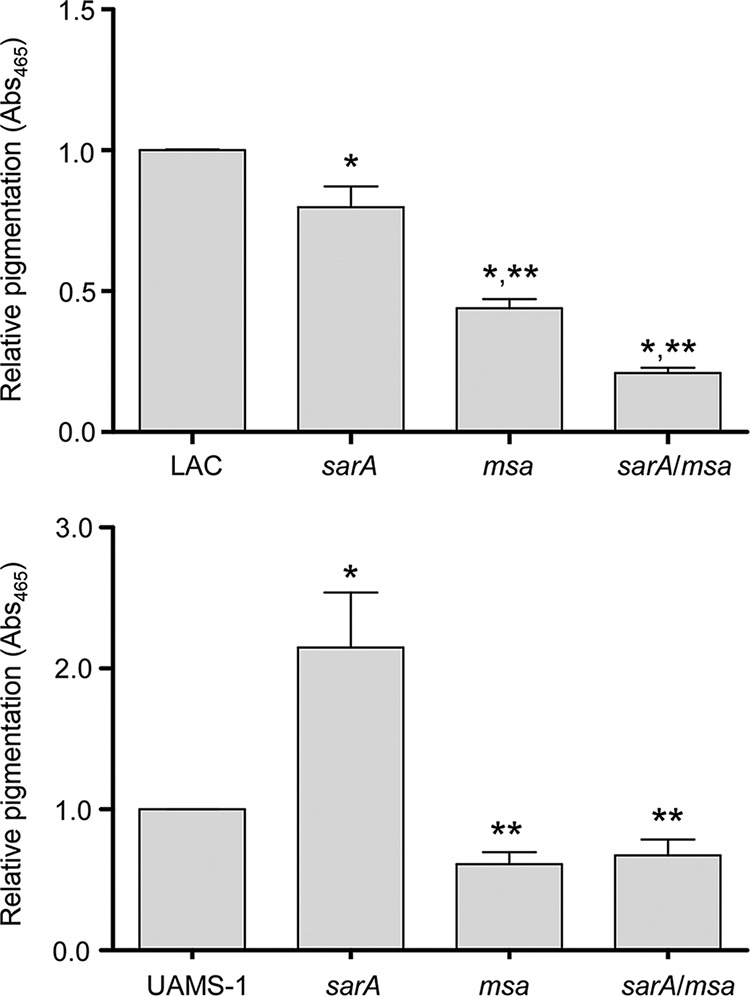
Staphyloxanthin production in *sarA* and *msa* mutants. Pigment was extracted from standardized samples of bacteria grown to stationary phase and measured at an absorbance of 465 nm. Bar charts represent cumulative results from at least four biological replicates, each of which included three experimental replicates. Error bars indicate standard errors of the means. *, statistical significance relative to the isogenic parent strain; **, statistical significance relative to the isogenic *sarA* mutants.

All of the results discussed above are consistent with a model in which *msa* functions upstream to enhance the production of SarA but also demonstrate that the impact of mutating *msa* on *sarA*-associated phenotypes is strain dependent. There are also reports implicating mutation of *msa* in LAC in phenotypes that have not been previously associated with *sarA*. One of these is that mutation of *msa* in LAC results in the reduced production of staphyloxanthin (27), which has been implicated as an important virulence factor in S. aureus (42). We examined this in LAC and UAMS-1 *sarA* and *msa* mutants, and the results confirmed that mutation of *msa* in LAC results in a statistically significant reduction in the production of staphyloxanthin (Fig. 10) and consequently reduced pigmentation of colonies on agar plates (data not shown). Importantly, unlike the relative impacts of mutating *sarA* and *msa* on biofilm formation and protease production, the impact of mutating *msa* exceeded that of mutating *sarA* in this regard, thus suggesting that the impact of mutating *msa* on staphyloxanthin production is primarily independent of its impact on *sarA*. In UAMS-1, the results of these assays provided an even more striking contrast. Specifically, staphyloxanthin production was increased in a UAMS-1 *sarA* mutant but decreased in the isogenic *msa* mutant (Fig. 10). Although the decrease observed in a UAMS-1 *msa* mutant was not statistically significant, this contrast nevertheless makes it evident that the impact of mutating *msa* on staphyloxanthin production in UAMS-1 is independent of its impact on *sarA*.

### Impact of *msa* in osteomyelitis.

**FIG 11:**
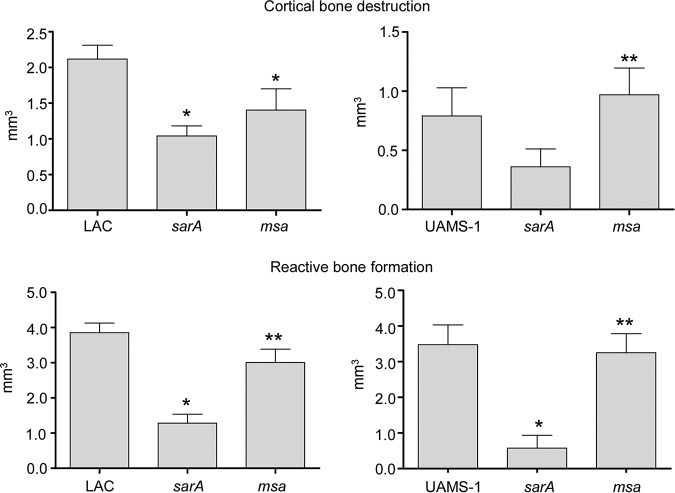
Impacts of *sarA* and *msa* on the virulence of LAC and UAMS-1 in an osteomyelitis model. Images were analyzed for cortical bone destruction and reactive (new) bone formation in C57BL/6 mice infected with LAC, UAMS-1, or their isogenic *sarA* and *msa* mutants. Values are presented as volumes relative to those in mock-infected mice which underwent the surgical procedure but were injected only with sterile PBS. At least ten mice were analyzed for each mutant or respective parent strain. *, statistical significance relative to the isogenic parent strain; **, statistical significance relative to the isogenic *sarA* mutant.

The results discussed above provide insight into the impact of *msa* on *sarA*-associated phenotypes in divergent clinical isolates of S. aureus. However, they also suggest, specifically, with respect to our staphyloxanthin assays, that *msa* serves regulatory functions that are independent of its impact on *sarA*. Moreover, all of these results are based on *in vitro* assays that do not necessarily reflect the unique microenvironment of the bone. Thus, we wanted to directly assess the relative contribution of *msa* and *sarA* to virulence in our murine osteomyelitis model (3, 43). As previously reported (3), mutation of *sarA* limited virulence in both strains as assessed by reactive bone formation and cortical bone destruction, although in this experiment, the reduction in cortical bone destruction observed with the UAMS-1 *sarA* mutant did not reach statistical significance (Fig. 11). By comparison, mutation of *msa* had only a modest impact on virulence in LAC, particularly in the context of cortical bone destruction, and it had no significant impact in UAMS-1 in either reactive bone formation or cortical bone destruction.

### Conclusions.

Most reports describing the impact of S. aureus regulatory loci on clinically relevant phenotypes, including virulence, are based on examination of single loci in a single strain, and this makes it difficult to reach conclusions regarding the relative potential of different regulatory loci as therapeutic targets. We have attempted to address this by directly comparing different regulatory mutants generated in divergent clinical isolates of S. aureus using both *in vitro* and *in vivo* assays (3, 4, 44). The results of these studies have led us to focus on *sarA* and to hypothesize that a primary factor contributing to the impact of mutating *sarA* on virulence and virulence-associated phenotypes is the increased production of extracellular proteases and the limitation this imposes on the accumulation of both surface-associated and extracellular virulence factors (1, 16). To date, we have not included the *msaABCR* operon in these studies, and it is important to do so given that *msa* has been shown to function upstream of *sarA* and to impact *sarA*-associated phenotypes, including biofilm formation and protease production (25–27, 29). This raises the possibility that *msa* could also be a viable therapeutic target. Experimentally addressing this possibility was the focus of the experiments we report. However, the results we report lead us to conclude that this is not the case for two reasons. First, even in the genetically and phenotypically divergent clinical isolates LAC and UAMS-1, the impacts of mutating *msa* on biofilm formation and virulence in our osteomyelitis model were limited in comparison to those of mutating *sarA*. Second, the relative impacts of mutating *msa* differed between these two strains with respect to both of these phenotypes. This emphasizes the need for direct comparative studies like those we report, particularly given the complexity of S. aureus regulatory circuits and the diversity among S. aureus strains as represented by the USA300 isolate LAC and the USA200 strain UAMS-1.

## MATERIALS AND METHODS

### Bacterial strains and growth conditions.

The strains used in these experiments are summarized in Tables 2 and 3. LAC and UAMS-1 mutants produced during the course of this work were generated by ϕ11-mediated transduction from existing mutants (1, 4, 13, 15, 27, 34, 44–53). Protease reporter plasmids were also introduced into the designated mutants by ϕ11-mediated transduction (23). All strains were maintained at −80°C in tryptic soy broth (TSB) containing 25% (vol/vol) glycerol. For each experiment, strains under study were retrieved from cold storage by plating on tryptic soy agar (TSA) with appropriate antibiotic selection. Antibiotics were incorporated into the culture media as appropriate at the following concentrations: chloramphenicol, 10 μg ml^−1^; kanamycin, 50 μg ml^−1^; neomycin, 50 μg ml^−1^; erythromycin, 10 μg ml^−1^; spectinomycin, 1 mg ml^−1^; and tetracycline 5 μg ml^−1^. Kanamycin and neomycin were always used together to avoid selection of spontaneously resistant strains.

**TABLE 2:** LAC S. aureus strains used in this study

Strain	Genotype and/or description	Reference
UAMS-2279^*a*^	Wild type	1
UAMS-2294	*sarA*::*kan neo*	1
UAMS-4001	*sarA*::*kan neo*, pSARA	1
UAMS-4520	Δ*msaABCR*	27
UAMS-4521	Δ*msaABCR*, pCN34::*msaABCR*	27
UAMS-4601	Δ*msaABCR*, pSARA	This work
UAMS-4545	Δ*msaABCR sarA*::*kan neo*	This work
UAMS-4222	Wild type, pCM13 (*aur*::*sgfp*)	23
UAMS-4223	*sarA*::*kan neo*, pCM13 (*aur*::*sgfp*)	This work
UAMS-4537	Δ*msaABCR*, pCM13 (*aur*::*sgfp*)	This work
UAMS-4226	Wild type, pCM15 (*spl*::*sgfp*)	23
UAMS-4227	*sarA*::*kan neo*, pCM15 (*spl*::*sgfp*)	This work
UAMS-4538	Δ*msaABCR*, pCM15 (*spl*::*sgfp*)	This work
UAMS-4230	Wild type, pCM16 (*ssp*::*sgfp*)	23
UAMS-4231	*sarA*::*kan neo*, pCM16 (*ssp*::*sgfp*)	This work
UAMS-4539	Δ*msaABCR*, pCM16 (*ssp*::*sgfp*)	This work
UAMS-4234	Wild type, pCM35 (*scp*::*sgfp*)	23
UAMS-4235	*sarA*::*kan neo*, pCM35 (*scp*::*sgfp*)	This work
UAMS-4446	*spa*::*erm*	34
UAMS-4552	*hla*::*erm*	52
UAMS-4540	Δ*msaABCR*, pCM35 (*scp*::*sgfp*)	This work
UAMS-3001	Δ*aur* Δ*sspAB* Δ*scpA spl*::*erm*	47
UAMS-3002	*sarA*::*kan neo* Δ*aur* Δ*sspAB* Δ*scpA spl*::*erm*	1
UAMS-4557	Δ*msaABCR* Δ*aur* Δ*sspAB* Δ*scpA spl*::*erm*	This work
UAMS-2280	*nuc*::*ltrB*	41
UAMS-2295	*sarA*::*kan neo nuc*::*ltrB*	This work
UAMS-4582	Δ*msaABCR nuc*::*ltrB*	This work

a Variant of the clinical isolate LAC which has been cured of the erythromycin resistance plasmid as previously described (1).

**TABLE 3:** UAMS-1 S. aureus strains used in this study

Strain	Genotype and/or description	Reference
UAMS-1	Wild type	48
UAMS-929	*sarA*::*kan neo*	30
UAMS-969	*sarA*::*kan neo*, pSARA::*cat*	30
UAMS-4499	Δ*msaABCR*	46
UAMS-4500	Δ*msaABCR*, pCN34::*msaABCR*	46
UAMS-4603	Δ*msaABCR*, pSARA	This work
UAMS-4549	Δ*msaABCR sarA*::*kan neo*	This work
UAMS-4220	Wild type, pCM13 (*aur*::*sgfp*)	This work
UAMS-4221	*sarA*::*kan neo*, pCM13 (*aur*::*sgfp*)	This work
UAMS-4541	Δ*msaABCR*, pCM13 (*aur*::*sgfp*)	This work
UAMS-4224	Wild type, pCM15 (*spl*::*sgfp*)	This work
UAMS-4225	*sarA*::*kan neo*, pCM15 (*spl*::*sgfp*)	This work
UAMS-4542	Δ*msaABCR*, pCM15 (*spl*::*sgfp*)	This work
UAMS-4228	Wild type, pCM16 (*ssp*::*sgfp*)	This work
UAMS-4229	*sarA*::*kan neo*, pCM16 (*ssp*::*sgfp*)	This work
UAMS-4543	Δ*msaABCR*, pCM16 (*ssp*::*sgfp*)	This work
UAMS-4232	Wild type, pCM35 (*scp*::*sgfp*)	This work
UAMS-4233	*sarA*::*kan neo*, pCM35 (*scp*::*sgfp*)	This work
UAMS-4544	Δ*msaABCR*, pCM35 (*scp*::*sgfp*)	This work
UAMS-321	*ica*::*tet*	49
UAMS-1624	*codY*::*ermC*	50
UAMS-4412	*xerC*::*erm*	51
UAMS-1471	Δ*nuc*	13
UAMS-1477	*sarA*::*kan neo* Δ*nuc*	13
UAMS-4556	Δ*msaABCR* Δ*nuc*	This work
UAMS-4574	Δ*aur* Δ*sspAB scpA*::*tet*	This work
UAMS-4578	*sarA*::*kan neo* Δ*aur* Δ*sspAB scpA*::*tet*	This work
UAMS-4583	*ΔmsaABCR* Δ*aur* Δ*sspAB scpA*::*tet*	This work

### Preparation of S. aureus conditioned medium.

To prepare conditioned medium (CM), cultures of each strain were grown overnight (16 h) in TSB at 37°C with constant shaking. The optical density at 560 nm (OD_560_) of each culture was determined, and fresh TSB was added to standardize each culture to an equivalent optical density. Cells were then removed by centrifugation and CM prepared by filter sterilization. Samples were stored at −80°C until used.

### Preparation of whole-cell lysates.

Whole-cell lysates were prepared as previously described with minor modification (45). Briefly, strains were cultured at 37°C in TSB with constant shaking and a medium-to-flask ratio of 0.5. Bacterial cells from a volume of each culture calculated to obtain an equivalent number of cells were harvested by centrifugation at an OD_560_ of approximately 1.5, 4.0, and 10.0, which correspond to the mid‐exponential, late‐exponential, and post‐exponential growth phases, respectively. Cells were washed with sterile phosphate-buffered saline (PBS) and resuspended in 750 μl of TEG buffer (25 mM Tris‐HCl [pH 8.0], 25 mM EGTA). Cell suspensions were stored at −20°C until all samples had been collected, at which point samples were thawed on ice, transferred to Fastprep Lysing Matrix B tubes, and lysed in a FastPrep-24 benchtop homogenizer (MP Biomedicals) using two 40-s intervals at a rate of 6.0 m/s interrupted by a 5-min interval during which the homogenates were chilled on ice. After centrifugation at 15,000 × *g* for 10 min at 4°C, supernatants were harvested and stored at −80°C.

### Western blotting.

SarA Western blotting was performed with an anti-SarA antibody and appropriate secondary antibodies, as previously described (1, 15, 16). Western blots included at least two biological replicates. Densitometric values were obtained with a Bio-Rad ChemiDoc MP imaging system and Image Lab software (Bio-Rad Laboratories).

### RNA isolation and real-time qPCR.

Overnight cultures of S. aureus were diluted 1:10 in fresh TSB and incubated at 37°C with shaking (200 rpm) for 2 h. The cells were then normalized to an OD_600_ of 0.05 in 25 ml TSB in a 125-ml conical flask and incubated at 37°C with shaking (200 rpm). The cells were collected at mid-exponential growth phase. Total RNA was isolated from cells using a Qiagen RNeasy Maxi column (Qiagen), as previously described (27). The quality of total RNA was determined by NanoDrop spectrometer readings, and 1 μg RNA was used to synthesize cDNA using iScript Reverse Transcription Supermix for reverse transcription-quantitative PCR (RT-qPCR) (Bio-Rad). RT-qPCR was performed using iTaq Universal SYBR Green Supermix (Bio-Rad) as described previously (27). The constitutively expressed gyrase A (*gyrA*) gene was used as an endogenous control gene and was included in all experiments. The following primer sequences were used to measure *sarA* expression: RT-*sarA*-F, TTTGCTTCAGTGATTCGTTTATTTACTC, and RT-*sarA*-R, GTAATGAGCATGATGAAAGAACTGTATT. Analysis of expression of each gene was conducted based on at least three biological replicates.

### Static *in vitro* biofilm assay.

Biofilm formation was assessed *in vitro* using a microtiter plate assay as previously described (28). Briefly, sterile 96-well microtiter plates were coated with 100 μl of 20% carbonate-bicarbonate–reconstituted human plasma (Sigma) and incubated overnight at 4°C. Bacterial cultures were grown overnight in TSB supplemented with 3% sodium chloride and 0.5% glucose (biofilm medium [BFM]) at 37°C. Cultures were standardized to an OD_560_ of 0.05 in fresh BFM. Plasma was gently aspirated, and the microtiter plate was inoculated with 200 μl of standardized culture per well. The plate was incubated statically overnight at 37°C. Wells were gently washed three times with 200 μl PBS, fixed with 200 μl 100% ethanol (EtOH), stained with 200 μl Gram’s crystal violet, and finally washed three times with 250 μl PBS. The stain was eluted with 100 μl 100% EtOH for 10 min, the eluent was diluted into a new 96-well plate, and the absorbance was measured at 595 nm with a FLUOstar Omega microplate reader (BMG Labtech).

### Total protease activity.

Total protease activity of CM was assessed using the FRET-based protease fluorescent detection kit (Sigma) and the EnzChek gelatinase/collagenase assay kit (Thermo Fisher Scientific), both according to the manufacturer’s instructions.

### Protease reporter assay.

Strains carrying each protease reporter (pCM13, pCM15, pCM16, or pCM35) were cultured in TSB overnight as detailed above. Cultures were then standardized to an OD_560_ of 10.0. Two hundred microliters of each standardized culture was then aliquoted in triplicates into a black clear-bottomed 96-well plate, and the mean fluorescence intensity (MFI) was measured with a FLUOstar Omega microplate reader (excitation, 485 nm; emission, 520 nm) (BMG Labtech).

### PIA immunoblot.

Production of the polysaccharide intercellular adhesin (PIA) was assessed as previously described with minor modifications (44). Specifically, cultures were grown overnight in BFM. After standardization to OD_560_ of 5.0, cells were harvested by centrifugation and resuspended in 60 μl 0.5 M EDTA. Cell suspensions were boiled for 5 min followed by centrifugation (14,000 × *g* for 2 min). Forty microliters of the supernatant was then incubated for 30 min at 48°C with 1 μl proteinase K (10 mg/ml). Twenty microliters of Tris-buffered saline (20 mM Tris-HCl, 150 mM NaCl [pH 7.4]) was added to each sample, which was then stored at −20°C. For analysis, 2 μl of each sample was spotted directly to a dry nitrocellulose membrane, and PIA was detected by using an anti-PIA antibody as previously described (44).

### Characterization of exoprotein profiles.

Exoprotein profiles were examined as previously described (1). CM harvested as described above was resolved by SDS-PAGE using 4% to 12% gradient Novex Bis-Tris Plus gels (Life Technologies). Proteins were visualized by staining with SimplyBlue SafeStain (Life Technologies). Images were obtained using a Bio-Rad ChemiDoc MP imaging system (Bio-Rad Laboratories).

### Staphyloxanthin production.

The relative production of staphyloxanthin was assessed using bacterial cells harvested from overnight cultures as previously described (27). Briefly, cells were harvested and standardized to an OD_560_ of 10.0 and washed twice with sterile water. Cells were then resuspended in 1.0 ml of 100% methanol and heated at 55°C for 5 min with occasional vortexing. The cells were removed by centrifugation at 15,000 × *g* for 1 min, and 100 μl of supernatant was placed into a 96-well microtiter plate in triplicates. Absorbance values were read on a FLUOstar Omega microplate reader (BMG Labtech) at 465 nm and background corrected with a methanol blank.

### Murine model of posttraumatic osteomyelitis.

The murine model of acute posttraumatic osteomyelitis was performed as previously described (43). Prior to surgery, 8- to 10-week-old C57BL/6 mice received 2.0 mg/kg of body weight meloxicam via subcutaneous injection and were then anesthetized with isoflurane for the duration of the surgery. For each mouse, an incision was made above the right hind limb. The periosteum was pulled apart with forceps, and using a 21-gauge Precision Glide needle (Becton, Dickinson), a 1-mm uni-cortical bone defect was made at the lateral midshaft of the femur. A bacterial inoculum of 1 × 10^6^ CFU in 2 μl of PBS was delivered into the intramedullary canal. The periosteum and skin were then closed with sutures, and the mice were allowed to recover from anesthesia. Infection was allowed to proceed for 14 days thereafter, at which time the mice were euthanized and the right femur was removed and subjected to micro-computed tomography (micro-CT) analysis. All experiments involving animals were reviewed and approved by the Institutional Animal Care and Use Committee of the University of Arkansas for Medical Sciences and were performed according to NIH guidelines, the Animal Welfare Act, and U.S. federal law.

### Micro-computed tomography.

The analysis of cortical bone destruction and new bone formation was performed using micro-CT imaging with a Skyscan 1174 micro-CT (Bruker), and scans were analyzed using the manufacturer’s analytical software. Briefly, axial images of each femur were acquired at a resolution of 6.7 μm at 50 kV and 800 μA through a 0.25-mm aluminum filter. Bones were visualized using a scout scan and then scanned in three sections as an oversize scan to image the entire femoral length. The volume of cortical bone was isolated in a semiautomated process per the manufacturer’s instructions. Briefly, cortical bone was isolated from soft tissue and the background by global thresholding (low threshold, 89; high threshold, 255). The processes of opening, closing, dilation, erosion, and despeckling were configured using the bones from sham-treated controls to separate the new bone from the existing cortical bone, and a task list was created to apply the same process and values to all bones in the data set. After processing of the bones using the task list, the volume of interest (VOI) was corrected by drawing inclusive or exclusive contours on the periosteal surface. Cortical bone destruction analysis consisted of approximately 1,800 slices between anatomical landmarks at opposing ends of the femur. Destruction was determined by subtraction of the volume of infected bones from the average bone volume from sham-treated controls. Reactive new bone formation was assessed by first isolating the region of interest (ROI) that contained only the original cortical bone (as described above). After cortical bone isolation, the new bone volume was determined by subtracting the cortical bone volume from the total bone volume. All calculations were performed on the basis of direct voxel counts.

### Statistical analysis.

To allow for statistical comparison across biological and experimental replicates, the results obtained for each experimental replicate with each strain were averaged across all biological replicates. For densitometric analyses of Western blots, protease assays, biofilm assays, and pigmentation assays, results observed with the isogenic wild-type strain were set to 1.0, and these averages were then plotted relative to the results observed with this strain. For protease reporter assays and micro-CT analysis, absolute values were plotted for all replicates obtained with each strain. Analysis of variance (ANOVA) models with Dunnett’s posttest adjustment were used to assess statistical significance. *P* values of ≤0.05 were considered to be statistically significant. Statistical analyses were performed using the statistical programming language R version 3.3.3 (R Foundation for Statistical Computing, Vienna, Austria), SAS 9.4 (SAS Institute Inc., Cary, NC), and GraphPad Prism 5.0 (GraphPad Software, La Jolla, CA).

## Supplementary Material

Supplemental file 1

Supplemental file 2

Supplemental file 3
