# Supplementary material for: The Impacts of msaABCR on sarA-Associated Phenotypes Are Different in Divergent Clinical Isolates of Staphylococcus aureus
Source: Infect Immun. 2020 Jan 22;88(2):e00530-19. doi: 10.1128/IAI.00530-19 (PMC6977130; doi:10.1128/IAI.00530-19)
Supplement: Supplemental file 1 [file IAI.00530-19-s0001.pdf]

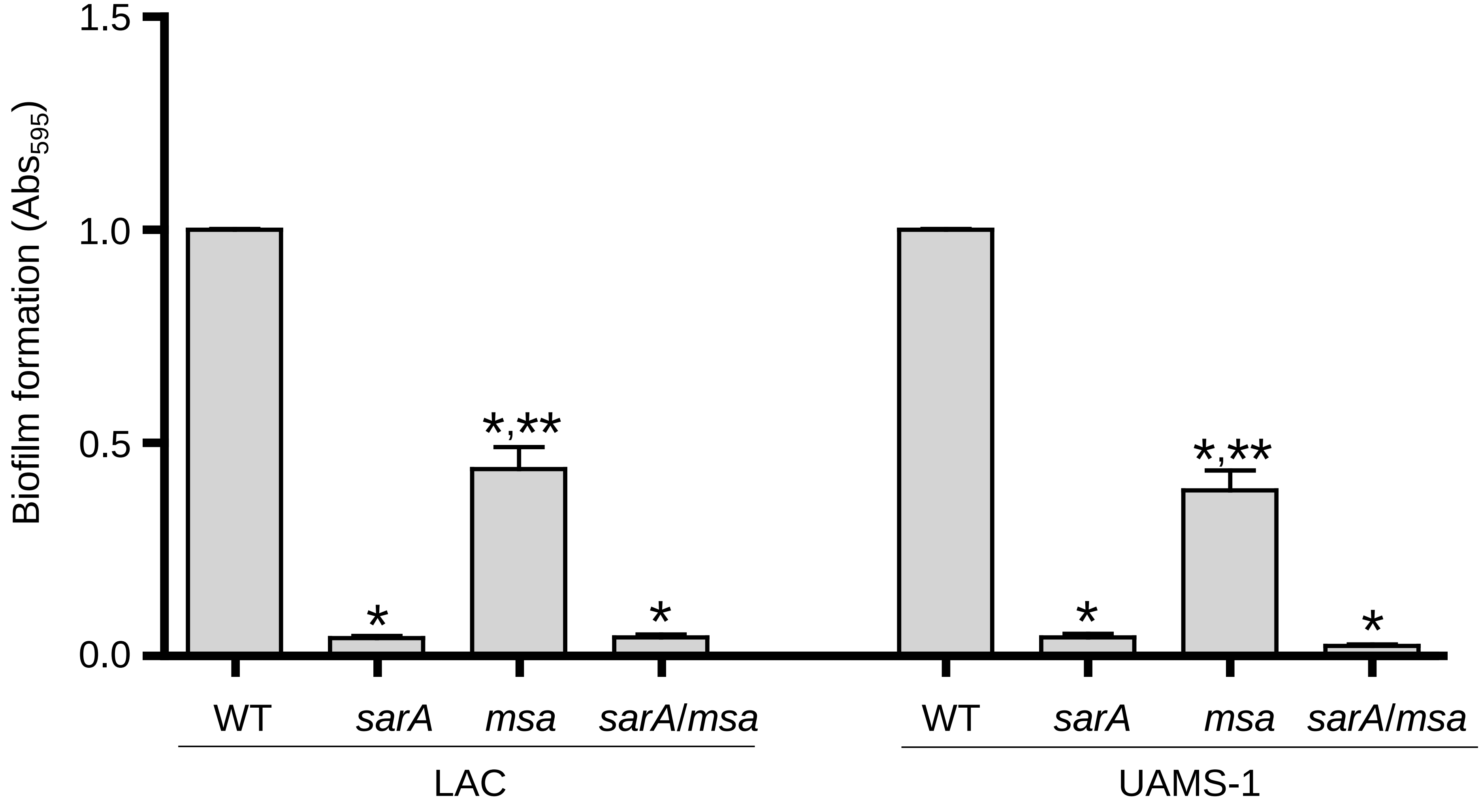

Suppl. Fig. 1. Relative impact of *msa* and *sarA* on biofilm formation. Biofilm formation was assessed with the wild-type (WT) strains LAC and UAMS-1 and isogenic *sarA, msa* and *sarA/msa* mutants of each. Error bars indicate standard error of the mean. Single asterisk indicates statistical significance relative to the isogenic parent strain. Double asterisks indicate statistical significance relative to the isogenic *sarA* mutant.
