# Supplementary material for: The Impacts of msaABCR on sarA-Associated Phenotypes Are Different in Divergent Clinical Isolates of Staphylococcus aureus
Source: Infect Immun. 2020 Jan 22;88(2):e00530-19. doi: 10.1128/IAI.00530-19 (PMC6977130; doi:10.1128/IAI.00530-19)
Supplement: Supplemental file 2 [file IAI.00530-19-s0002.pdf]

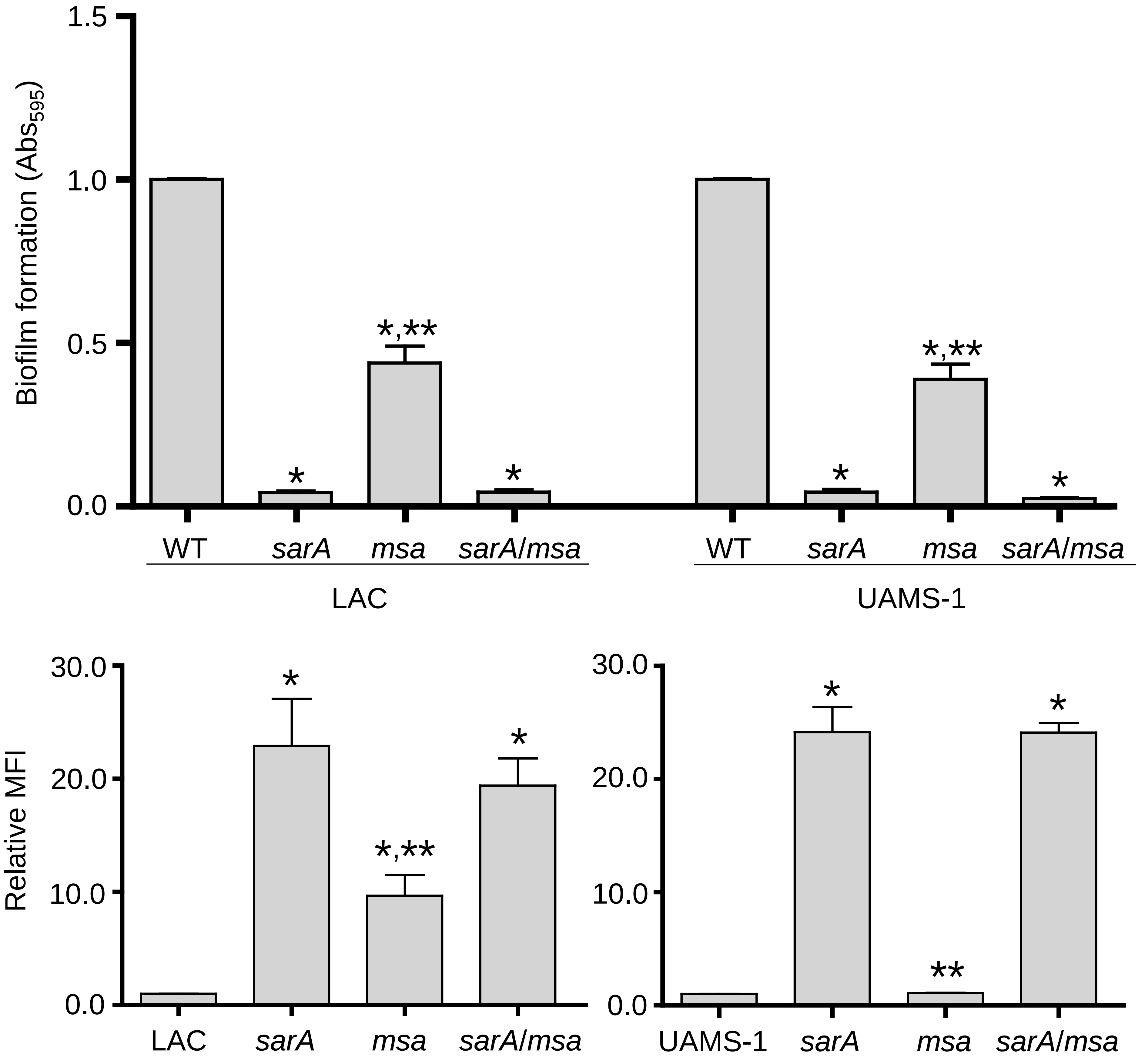

Suppl. Fig. 2. Inverse correlation between protease production and biofilm formation in LAC *msa* and *sarA* mutants. Biofilm formation was assessed with the wild-type (WT) strains LAC and UAMS-1 and their isogenic *sarA*, *msa* and *sarA/msa* mutants (top). Protease activity was assessed with CM from the same strains using an FITC-gelatin cleavage hydrolysis assay (bottom). Bar charts are representative of results from at least two biological replicates, each of which included three experimental replicates. Error bars indicate standard error of the mean. In both panels, single asterisk indicates statistical significance relative to the isogenic parent strain. Double asterisks indicate statistical significance relative to the isogenic *sarA* mutant.
