# Supplementary material for: The Impacts of msaABCR on sarA-Associated Phenotypes Are Different in Divergent Clinical Isolates of Staphylococcus aureus
Source: Infect Immun. 2020 Jan 22;88(2):e00530-19. doi: 10.1128/IAI.00530-19 (PMC6977130; doi:10.1128/IAI.00530-19)
Supplement: Supplemental file 3 [file IAI.00530-19-s0003.pdf]

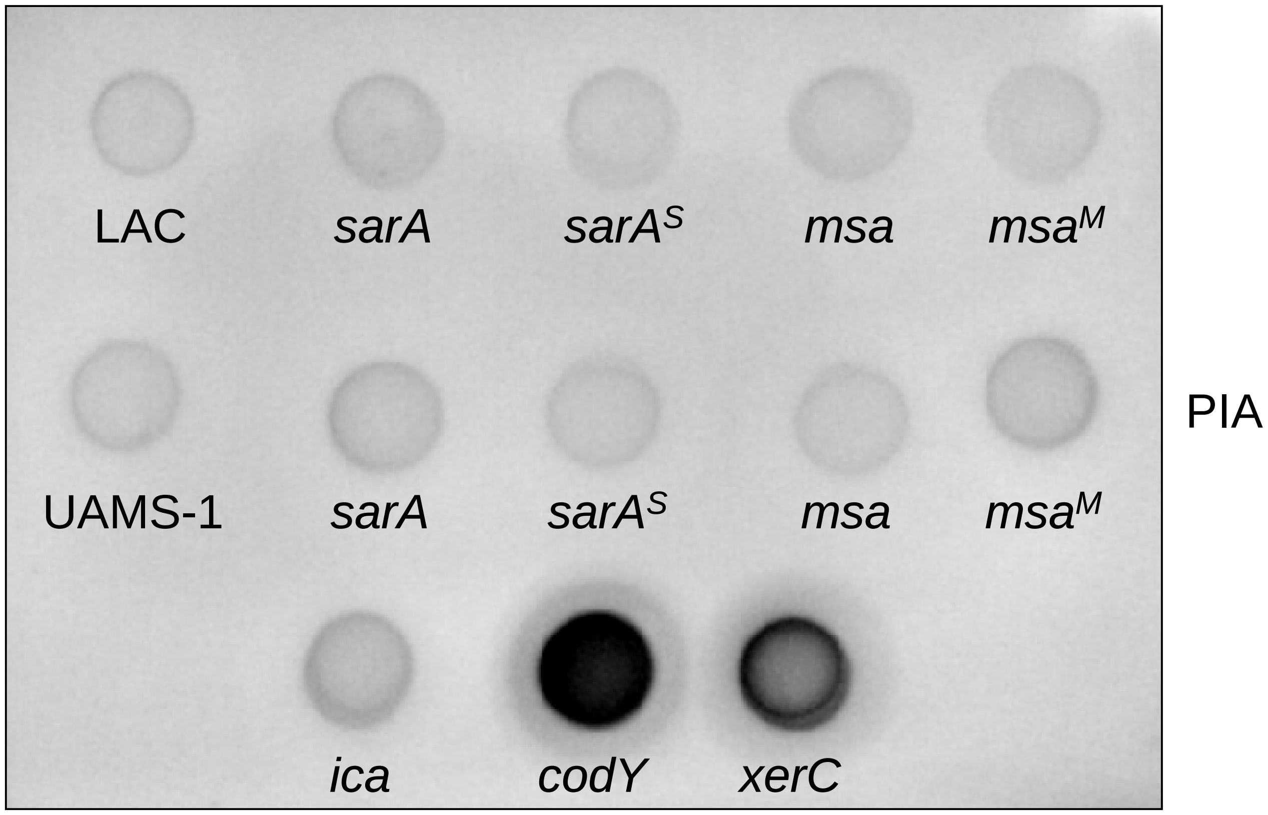


**Suppl Fig. 3. Impact of *sarA* nor *msa* on PIA production.** PIA production was assessed by dot blot using antibodies specific for PIA. A UAMS-1 isogenic *ica* mutant was included as a negative control. UAMS-1 *codY* and *xerC* mutants were included as positive controls based on our previous report (4).
